# 142. Impact of an Antimicrobial Stewardship Intervention on Antibiotic Prescribing in Patients with Obstetric Infection and Penicillin Allergy

**DOI:** 10.1093/ofid/ofab466.142

**Published:** 2021-12-04

**Authors:** Katelyn Quartuccio, Kelly Golden, Brenda L Tesini, Eric Heintz, Neil Seligman, Jessica Stern

**Affiliations:** 1 University of Rochester Medical Center, Highland Hospital, Rochester, NY; 2 Highland Hospital, Rochester, New York; 3 New York Emerging Infections Program University of Rochester, Rochester, NY; 4 University of Rochester Medical Center, Rochester, New York; 5 University of Rochester Medical Center- Strong Memorial Hospital, Rochester, New York

## Abstract

**Background:**

Antibiotics are commonly administered in the peripartum period and most patients with penicillin allergy can tolerate beta lactams, which are preferred for the prophylaxis and treatment of several common obstetric infections. The purpose of this study was to evaluate the impact of a stewardship intervention bundle (including updates to institutional antibiotic guidelines, reclassification of severe penicillin allergy, development of order sets, and a physician champion) on the management of obstetric infections in patients with reported penicillin allergy.

**Methods:**

This was a multicenter, retrospective study of adult patients presenting for labor and delivery who received at least one dose of antibiotics for an infectious indication May 1, 2018 to October 31, 2018 (pre-intervention) and May 1 2020 to October 31, 2020 (post-intervention). The primary outcome was the composite rates of patients with a reported penicillin allergy who received a preferred agent for Group B *Streptococcus* (GBS) prophylaxis, intraamniotic infection, or cesarean surgical site infection (SSI) prophylaxis.

**Results:**

A total of 192 patients with a documented penicillin allergy were evaluated (96 patients each in pre- and post-intervention groups). Hives were the most commonly reported allergy in both groups (40% vs 39%, P=0.883). Following stewardship interventions, there was a significant increase in the rate of preferred antibiotics prescribed to patients with penicillin allergy (34.3% vs 84.3%, P< 0.001), driven mainly by patients with non-severe allergy (18.4% vs 82.9%, P< 0.001). There were non-statistically significant trends toward lower rates of postpartum endometritis, 30-day readmission, 90-day SSI, and neonatal early onset sepsis. Allergic reactions in the post-intervention group were limited to itching and rash in one patient each; both resolved with medical management.

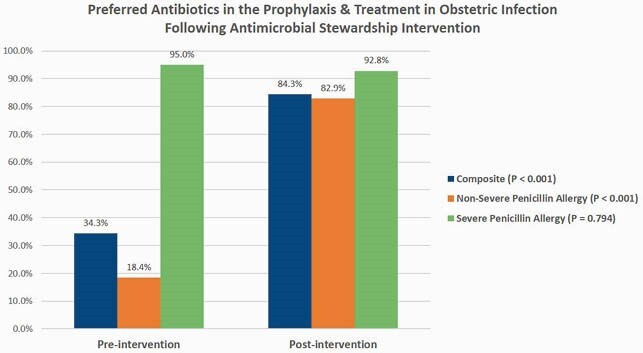

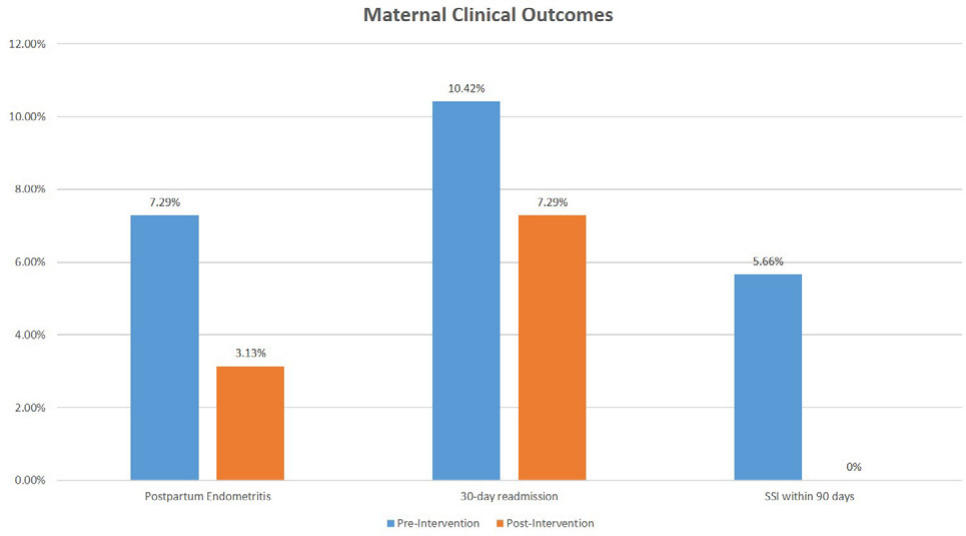

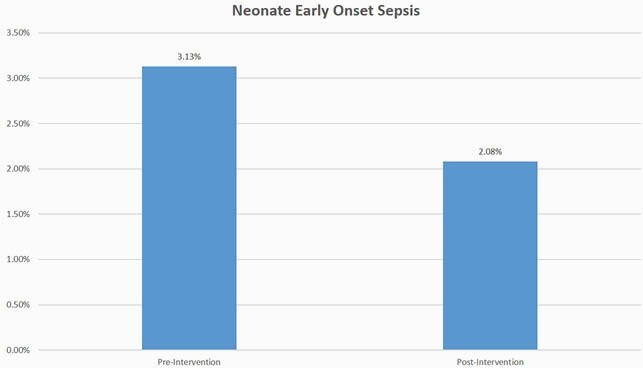

**Conclusion:**

A comprehensive antibiotic stewardship intervention increased preferred antibiotic prescribing for treatment and prophylaxis of obstetric infections. Pregnant patients with non-severe penicillin allergies, even those reporting hives, can tolerate beta-lactam antibiotics. The potential positive impact on clinical outcomes warrants additional investigation.

**Disclosures:**

**Neil Seligman, MD**, **Natera** (Consultant)**UpToDate** (Other Financial or Material Support, Author)

